# Mesenchymal stem cells protect against the tissue fibrosis of ketamine-induced cystitis in rat bladder

**DOI:** 10.1038/srep30881

**Published:** 2016-08-02

**Authors:** Aram Kim, Hwan Yeul Yu, Jinbeom Heo, Miho Song, Jung-Hyun Shin, Jisun Lim, Soo-Jung Yoon, YongHwan Kim, Seungun Lee, Seong Who Kim, Wonil Oh, Soo Jin Choi, Dong-Myung Shin, Myung-Soo Choo

**Affiliations:** 1 Department of Urology, Asan Medical Center, University of Ulsan College of Medicine, Seoul, 05505, Korea; 2 Department of Biomedical Sciences, Asan Medical Center, University of Ulsan College of Medicine, Seoul, 05505, Korea; 3 Department of Physiology, University of Ulsan College of Medicine, Seoul, 05505, Korea; 4 Department of Biochemistry and Molecular Biology, University of Ulsan College of Medicine, Seoul, 05505, Korea; 5 Biomedical Research Institute, MEDIPOST Co., Ltd., Seongnam-si, Gyeonggi-do, 13494, Korea

## Abstract

Abuse of the hallucinogenic drug ketamine promotes the development of lower urinary tract symptoms that resemble interstitial cystitis. The pathophysiology of ketamine-induced cystitis (KC) is largely unknown and effective therapies are lacking. Here, using a KC rat model, we show the therapeutic effects of human umbilical cord-blood (UCB)-derived mesenchymal stem cells (MSCs). Daily injection of ketamine to Sprague-Dawley rats for 2-weeks resulted in defective bladder function, indicated by irregular voiding frequency, increased maximum contraction pressure, and decreased intercontraction intervals and bladder capacity. KC bladders were characterized by severe mast-cell infiltration, tissue fibrosis, apoptosis, upregulation of transforming growth factor-β signaling related genes, and phosphorylation of Smad2 and Smad3 proteins. A single administration of MSCs (1 × 10^6^) into bladder tissue not only significantly ameliorated the aforementioned bladder voiding parameters, but also reversed the characteristic histological and gene-expression alterations of KC bladder. Treatment with the antifibrotic compound N-acetylcysteine also alleviated the symptoms and pathological characteristics of KC bladder, indicating that the antifibrotic capacity of MSC therapy underlies its benefits. Thus, this study for the first-time shows that MSC therapy might help to cure KC by protecting against tissue fibrosis in a KC animal model and provides a foundation for clinical trials of MSC therapy.

Ketamine, a non-competitive N-methyl-D-aspartic acid (NMDA) receptor antagonist, has been used as a general anesthetic for many years. Because it has been abused as a recreational drug, the numbers of ketamine abusers have greatly increased in recent years^1^. Long-term abuse of ketamine can markedly affect the urinary system, causing lower urinary tract symptoms that resemble those of interstitial cystitis (IC), such as frequency, urgency, suprapubic pain, and hematuria^2^,^3^. Ketamine-induced cystitis (KC) is also associated with reduced bladder compliance, detrusor overactivity, low bladder capacity, hydronephrosis, and impaired renal function^4^.

As with IC, the cause of KC is not fully understood. Thus, few treatments can effectively relieve the symptoms of KC. In particular, ketamine abusers exhibit the same characteristic cystoscopic findings and histological changes in bladder biopsies as patients with IC^1^, which include a thinner urothelium, mast-cell infiltration, tissue inflammation, and fibrosis^5^,^6^. Ketamine and its metabolites might have a direct toxic effect on the urinary tract, causing chronic submucosal, detrusor muscle inflammation, and dysfunction in the epithelial permeability barrier of the bladder, thereby leading to IC-like symptoms^7^,^8^. Several studies have suggested potential treatments for KC, which have also been used for IC, such as medications (analgesics, antimuscarinics, antibiotics), hydrodistention, and transcauterization^9^,^10^. However, only a few treatments have been reported and a curative treatment is still lacking.

Recently, administration of mesenchymal stem/stromal cells (MSCs) was found to stimulate the regeneration of damaged tissues and secrete growth factors and cytokines that promote angiogenesis and cell survival and prevent apoptosis of damaged tissues in several intractable disorders^11^,^12^,^13^,^14^,^15^,^16^. MSCs are adult multipotent progenitor cells derived from a variety of adult tissues (eg, bone marrow, adipose, peripheral blood, and dental pulp) and fetal ones (eg, umbilical cord blood [UCB], Wharton’s jelly, placenta, and amniotic fluid)^13^,^14^,^15^,^17^,^18^. They can differentiate into several lineages (osteoblasts, chondrocytes, and adipocytes), and potentially other lineages including epithelial cells. They also display immunomodulatory, antioxidative, vasculature-protective, and antifibrotic properties due to the secretion of several paracrine factors^18^,^19^,^20^,^21^,^22^,^23^,^24^,^25^,^26^. Indeed, in several preclinical and clinical studies, stem cell therapy helped to treat a number of bladder disorders^27^,^28^,^29^.

We recently reported that therapy with human UCB-derived MSCs can successfully alleviate IC using a hydrochloride-induced IC animal model^6^. By differentiation into epithelial and stromal cells in the IC-injured bladders, the administered MSCs improved bladder voiding function and ameliorated the pathological characteristics of IC, including epithelial denudation, inflammation, mast-cell infiltration, abnormally increased neurofilament levels, and angiogenesis^6^. These observations led us to investigate whether MSCs could also improve the IC-like symptoms of KC, which is still considered an incurable disease. Using a rat KC animal model, we provide, for the first time, experimental evidence that MSC therapy also helps to reverse the IC-like symptoms of KC.

## Results

### Evaluation of bladder function using cystometry

**Figure 1:**
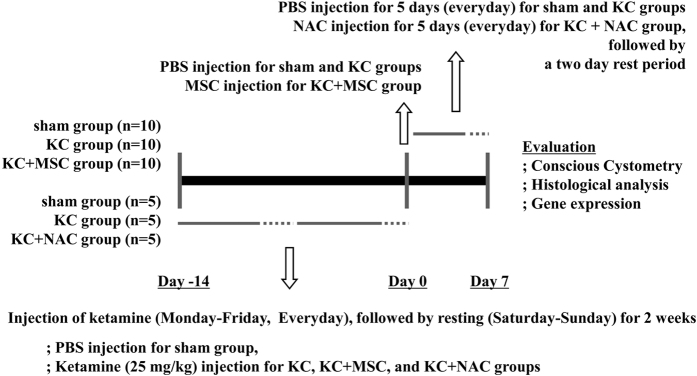
Schematic diagram of the study design. Ten or five female Sprague-Dawley rats were used in each group. The experimental control (KC group) had daily intravenous injection of ketamine for 5 days (solid line) followed by a 2-day rest period (dotted line) in each of two cycles. Interventions involved a single administration of human UCB-derived MSCs at a dose of 1 × 10^6^ cells per 200 μl PBS into the submucosal layer of the bladder (KC + MSC group) or daily intraperitoneal injection of 200 mg/kg NAC (KC + NAC group) at the indicated schedules. The sham group received PBS vehicle instead of MSC or NAC injection.

**Figure 2:**
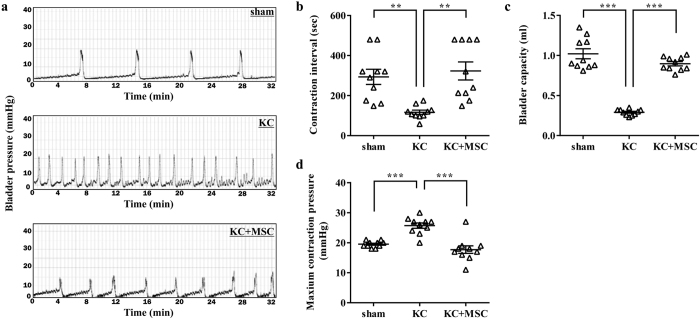
Administration of MSCs improves the voiding function of rats with KC. (**a**) Representative cystometry results of the indicated groups. (**b–d**) The contraction intervals (**b**), bladder capacity (**c**), and maximum contraction pressure (**d**) were quantified from the voiding pattern analysis. All data are presented as dot plot with the mean ± SEM. *n* = 10 **p < 0.01, ***p < 0.001 when the groups were compared by one-way analysis of variance with Bonferroni post-test.

We recently reported that daily injection of rats with ketamine for 2 week can induce the pathology seen in the KC bladder^30^. Using this KC animal model, we first examined whether MSC transplantation could ameliorate the defective voiding function of the KC bladder by performing conscious cystometric analysis, which monitors the ability of the bladder to contract and expel urine (Fig. 1). In agreement with our previous findings^30^, rats daily injected with ketamine at the dosage of 25 mg/kg for 2 weeks (KC group) exhibited increased and irregular voiding frequencies and decreased intercontraction intervals compared with sham group rats (118.8 ± 34.3 vs 306.5 ± 138.5 s, respectively; p = 0.0341; Fig. 2a,b). A single administration of 1 × 10^6^ MSCs into the submucosal layer of the bladder tissues (KC + MSC group) significantly improved the intercontraction interval compared with the KC group (339.0 ± 131.2 vs 118.8 ± 34.3 s, respectively; p = 0.0020). The bladder capacity of the KC group rats was decreased compared with that of the sham group rats (0.29 ± 0.03 vs 0.89 ± 0.09 mL, respectively; p < 0.0001). However, this defect was significantly reversed by the administration of MSCs (Fig. 2c). In addition, the KC group exhibited significantly higher maximum contraction pressure values than the normal control rats (27.6 ± 6.8 vs 21.5 ± 5.6 mmHg, respectively; p = 0.0208; Fig. 2d). Injection of MSCs into the bladder significantly reversed this effect compared with the KC-only rats (18.5 ± 1.0 vs 27.6 ± 6.8 mmHg, respectively; p = 0.0001). Taken together, these results show that a single administration of MSCs into the bladder can repair the damaged voiding function observed in the KC bladder.

### Histological analysis of the effect of MSC therapy

**Figure 3:**
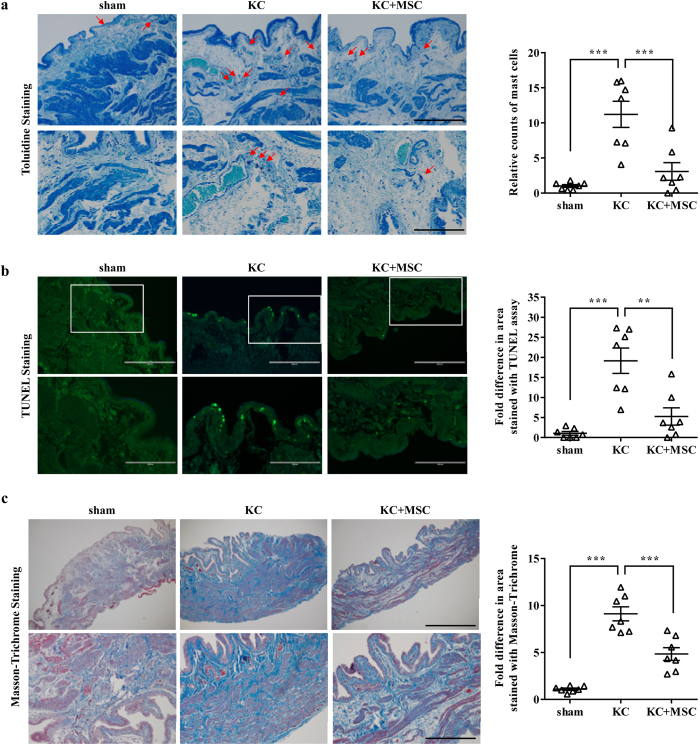
Histological analysis of the beneficial effects of MSCs on ketamine-induced bladder injuries. (**a**) The infiltrated mast-cells in bladder tissues were stained with Toluidine blue (red arrows in the left panels) and counted (right panel). The upper and lower images are at ×100 (scale bar = 400 μm) and ×200 (scale bar = 200 μm) magnification, respectively. (**b**) The apoptotic cells (green) in the bladder sections were stained with a TUNEL assay (left panel) and quantified (right panel). The region enriched in apoptotic cells (box in upper image; magnification ×100, scale bar = 400 μm) is shown in the lower panel at higher magnification (×200, scale bar = 200 μm). (**c**) Fibrosis in the bladder sections was stained with Masson’s trichrome stain (left panels). The blue color indicates fibrosis. The upper and lower images are at ×40 (scale bar = 1 mm) and ×100 (scale bar = 400 μm) magnification, respectively. Fibrosis was quantified by digital image analysis (lower panel). KC = ketamine; KC+MSC = ketamine-injected rats treated with MSC. All quantitative data were normalized to those of the sham group and are presented as dot plot with the mean ± SEM. *n* = 7, **p < 0.01, ***p < 0.001 compared with the KC group with Bonferroni post-test.

In line with clinical studies^31^,^32^, the bladders in the KC rat model are characterized by increased mast-cell infiltration, tissue apoptosis, and tissue fibrosis^30^. As previously reported, the bladders in the KC group exhibited markedly increased infiltration of Toluidine blue-stained mast-cells (Fig. 3a) and a higher number of terminal dUTP nick-end labeling (TUNEL)-stained apoptotic cells (Fig. 3b) compared with the sham group. MSC administration significantly reversed the infiltration of mast-cells and the apoptosis detected in the KC bladder (Fig. 3a,b). Furthermore, MSC therapy helped to prevent bladder tissue fibrosis (Fig. 3c), which is generally considered to be an important feature of KC^31^,^32^. Based on gross histological examination, severe denudation of the urothelium and inflammation were scarcely found in the bladders tested in all of the groups (Supplementary Fig. 1). Taken together, these results show that a single injection of MSCs into the bladder helps to treat the KC bladder by protecting against an abnormal alteration in mast-cell infiltration, apoptosis, and fibrotic damage.

### Engraftment of the administered MSCs in the KC bladder

**Figure 4:**
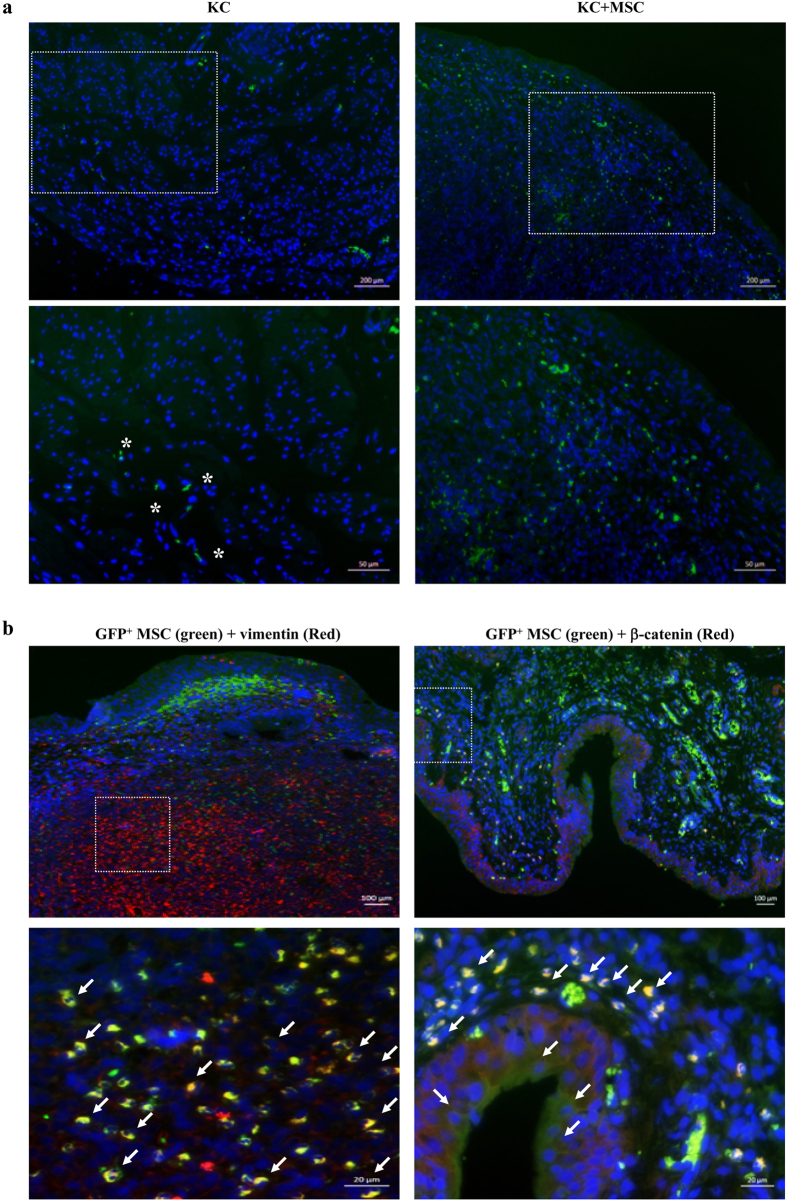
Engraftment of administered MSCs. (**a**) Fluorescent immunohistochemical detection of GFP-expressing MSCs in the bladder 7 day after the administration of MSCs stably expressing GFP. The region characterized by GFP^+^ cells (box in upper image; magnification ×100, scale bar = 200 μm) is shown in the lower panel at higher magnification (×200, scale bar = 50 μm). Nuclei were stained with DAPI (blue). * Indicates the auto-fluorescence signal from DAPI^-^ red blood cells. (**b**) Colocalization of GFP^+^ cells with vimentin^+^ stromal (left panel) and β-catenin^+^ urothelium (right panel) tissue in the KC bladder tissues. The upper and lower panel images are magnified ×100 (scale bar = 100 μm) and ×400 (scale bar = 20 μm), respectively. Arrow indicates the differentiated cells.

To examine the engraftment and fate of MSCs after injection, we tracked the administered MSCs, which stably expressed green fluorescent protein (GFP). As shown in Fig. 4a, GFP^+^ cells were mainly localized to the lamina propria and partly to the urothelium and, to a lesser extent, to the muscular layer. The GFP^+^ cells in the lamina propria and urothelium were costained with vimentin (a stromal cell marker) and β-catenin (an epithelial cell marker), respectively (Fig. 4b). These results indicate that the administered MSCs are well engrafted in the lamina propria near the basal layer of the urothelium in bladders for at least 1 week and that they can directly differentiate into stromal and urothelial cells.

### MSC therapy prevented the tissue fibrosis of the KC bladder

**Figure 5:**
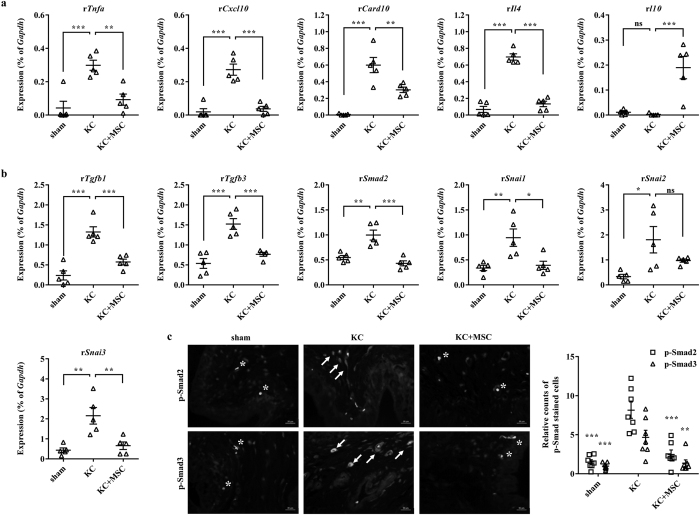
MSC therapy protects against fibrotic changes to the KC bladder. (**a,b**) RQ-PCR analysis of inflammation (**a**) and fibrosis (**b**)-related genes in the indicated bladder tissues. Expression is presented as % *Gapdh* and shown as dot plot with the mean ± SEM (*n* = 5; *p < 0.05, **p < 0.01, ***p < 0.001 compared with the KC group). ns = non-significant. (**c**) Fluorescent immunohistochemical detection of phosphorylated Smad2 or Smad3 protein (green) in the indicated bladder tissues (magnification ×400). Nuclei were stained with DAPI (blue). Scale bar = 20 μm. Asterisk and arrow indicate the non-specific auto-fluorescence and intra-nuclear staining of phosphorylated Smad proteins, respectively. Quantitative data were normalized to those of the sham group and are presented as dot plot with the mean ± SEM. *n* = 7, **p < 0.01, ***p < 0.001 compared with the KC group with Bonferroni post-test.

To understand the mechanism(s) underlying the benefits of MSC therapy, we examined the expression of genes related to inflammation, apoptosis, and tissue fibrosis by performing real-time quantitative-PCR (RQ-PCR) analysis. In accordance with the aforementioned histological alterations, the bladder tissues in the KC group exhibited higher levels of transcripts involved in inflammation (eg, tumor necrosis factor alpha [*Tnf-α*], chemokine C-X-C motif ligand 10 [*Cxcl10*], and interleukin 4 [*Il4*]) and apoptosis (eg, caspase recruitment domain family member 10 [*Card10*]) than the sham group (Fig. 5a). Upregulation of these genes was markedly inhibited in the bladder tissues of the KC+MSC group. Similarly, the expression of interleukin 10 (*Il10*), an anti-inflammatory cytokine^33^, was markedly upregulated in the bladder tissues of the KC + MSC group (Fig. 5a), suggesting that a single administration of MSCs could help to mitigate the inflammation and apoptosis induced by KC injury.

Our gene expression analysis also indicated that several genes related to tissue fibrosis, including transforming growth factor-beta 1 and -3 (*Tgf-β1* and *-β3*), SMAD family member 2 (*Smad2*), and snail family zinc finger 1, -2, and -3 (*Snai1*, *Snai2*, and *Snai3*), were markedly upregulated in the KC bladder. However, their expression was significantly suppressed by the administration of MSCs (Fig. 5b and Supplementary Fig. 2). Because fibrotic tissues are characterized by the activation of TGF-β signaling^34^, we examined the levels of phosphorylated Smad2 and Smad3 proteins, surrogate markers of activated TGF-β signaling (Fig. 5c). In line with the gene expression results, nuclear staining of phosphorylated Smad2 and Smad3 proteins was only observed in the bladders of the KC group, and not in the bladders of the KC + MSC and sham groups. Taken together, these results indicate that MSC administration helps to repress the fibrotic damage seen in the KC bladder.

### Antifibrotic therapy can effectively treat the KC bladder

**Figure 6:**
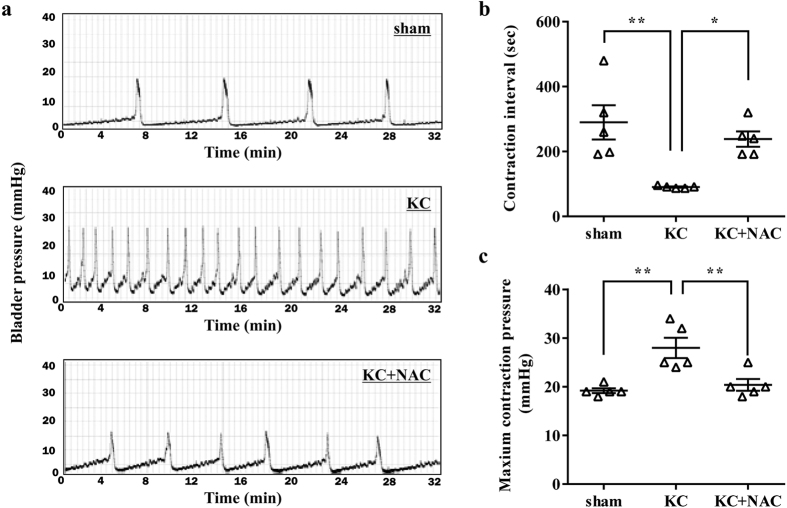
Pharmacological intervention with NAC improves bladder function in the KC rat group. (**a**) Representative cystometry results of the indicated groups. (**b,c**) Contraction intervals (**b**) and maximum contraction pressure (**c**) were quantified from voiding pattern analysis. All data are presented as dot plot with the mean ± SEM. *n* = 5, *p < 0.05, **p < 0.01 when the groups were compared by one-way analysis of variance with Bonferroni post-test.

**Figure 7:**
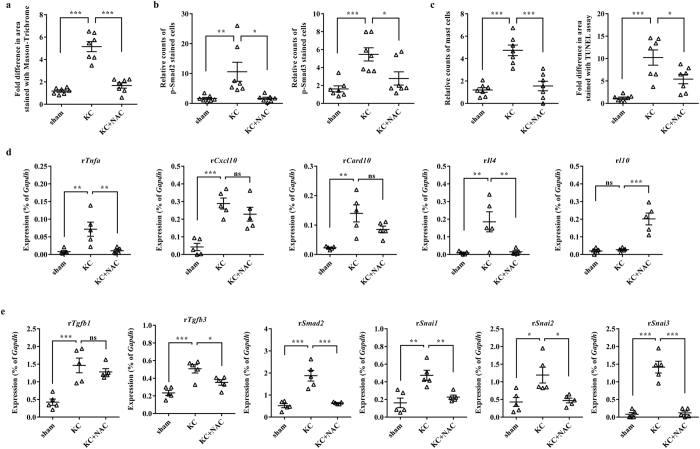
Histological and gene expression analysis of NAC pharmacologic therapy in the KC bladder. (**a–c**) Histological analysis of fibrosis (**a**), TGF-β activation (**b**), mast-cell infiltration and apoptosis (**c**) in the bladder tissues of the indicated groups. All quantitative data were normalized to those of the sham group and are presented as dot plot with the mean ± SEM. *n* = 7, *p < 0.05, **p < 0.01, ***p < 0.001 compared with the KC group with Bonferroni post-test. The representative photomicrographs of the above staining results are shown in Supplementary Figs S3 and S4. (**d,e**) RQ-PCR analysis of inflammation (**d**) and fibrosis (**e**)-related genes in the indicated bladder tissues. Expression is presented as % *Gapdh* and shown as dot plot with the mean ± SEM (*n* = 5; *p < 0.05, **p < 0.01, ***p < 0.001 compared with the KC group). ns = non-significant.

To better determine the role of antifibrotic activity in treating the KC bladder, we examined whether a pharmacological therapy with an antifibrotic compound such as N-acetylcysteine (NAC) could alleviate the symptoms and pathological characteristics of the KC bladder. NAC is reported to act as a scavenger of oxygen-free radicals and to directly alter the structure of TGF-β, thereby attenuating its pro-fibrotic activity^35^. Indeed, the use of NAC has shown benefits in patients with idiopathic pulmonary fibrosis by favorably altering the oxidative state of the lung^36^. Similar to MSC administration, daily intra-peritoneal injection of NAC at 200 mg/kg for 5 d significantly improved the defective voiding function observed in the bladders of the KC group (Fig. 6). As expected, NAC treatment significantly protected against the fibrotic change (Fig. 7a and Supplementary Fig. 3a) and TGF-β activation (Fig. 7b and Supplementary Fig. 3b) induced in the KC bladder. Notably, NAC therapy effectively reversed most of the characteristic pathophysiological features of the KC bladder, including mast-cell infiltration and apoptosis (Fig. 7c and Supplementary Fig. 4a,b). Accordingly, the altered expression of the inflammation- and fibrosis-related genes was blocked by daily injection of NAC (Fig. 7d). Taken together, these results show the significance of the antifibrotic ability of both MSCs and chemical-based approaches for treating the KC bladder.

## Discussion

The present study provides original preclinical data on the therapeutic potency of MSC therapy and the underlying mechanisms of the amelioration of the characteristic tissue injuries of the KC bladder. To the best of our knowledge, this study is the first to suggest an effective therapeutic approach for KC and ameliorating the crucial pathophysiological features of KC, including the abnormal mast-cell activation, apoptosis, and tissue fibrosis of the bladder.

KC is a recently identified condition that can have a severe and potentially long-lasting impact on the individual. Importantly, how ketamine damages the urinary tract is not clear, although several pathophysiological mechanisms have been suggested^7^. High concentrations of ketamine and/or its metabolites in the urine may have a direct toxic effect on the urothelium^37^ or induce acute papillary necrosis^7^, ultimately leading to an abnormal inflammatory response, apoptosis, and tissue fibrosis^38^. The obscure cause of KC hinders the development of effective curative strategies and the only known effective treatment is to abstain from the drug, but even that approach may not reverse the symptoms^39^. In this study, using an animal model, we prove that a single administration of MSCs is enough to improve the bladder voiding function (Fig. 2) and to prevent the pathological features (Figs 3 and 4) of the rat KC bladder. In particular, the MSC therapy effectively protected against the tissue fibrosis and activation of the TGF-β signaling pathway in the KC bladder (Fig. 5). The significance of the antifibrotic mechanism was further validated by the ability of NAC administration to also cure the KC (Figs 6 and 7). To successfully translate this promising preclinical study into clinical practice, efforts should be made to optimize the stem cell therapy protocols, such as the dosage and injection route, and to identify suitable stem cell sources in the clinical situation. In addition, combination therapy of MSCs and anti-fibrotic agents such as NAC could improve the therapeutic potency with minimizing the required number of stem cells, which could in turn lead to higher possibility of clinical applications of stem cell therapy for the treating of KC.

In the first report by Shahaniet *et al.*^40^ on KC, nine dependent ketamine users showed increased frequency and urgency of urination, dysuria, urge incontinence, and occasionally painful hematuria. Moreover, all patients had severe ulcerative cystitis in cystoscopy examination and four showed the presence of denuded urothelial mucosa with thin layers of reactive and regenerating epithelial cells and ulcerations with vascular granulation tissue and scattered inflammatory cells. According to our previous study, the hydrochloride-induced IC rat model is characterized by severe urothelial inflammation and denudation^6^. In contrast, in our KC animal model, the gross histological examination indicated a less significant urothelial junction protein defect and epithelial denudation (Supplementary Fig. 1). Indeed, several previous studies have reported contradictory results regarding the induction of urothelial degeneration in the bladders of ketamine-treated rats^41^,^42^,^43^. These contradictory results are likely due to the different protocols used to induce KC injury. Thus, the dose and duration of ketamine in our study might not have been sufficient to directly damage the urothelium. Accordingly, the establishment of an orthodox animal model that could precisely recapitulate the pathological conditions of KC patients should be considered to aid in the development of the most effective stem cell therapeutic approach for KC.

Mechanistically, MSC therapy exerted its beneficial effects by ameliorating the fibrotic damage, as well as reducing the abnormal infiltration of mast-cells and apoptosis, of the KC bladder (Figs 3 and 4). The significance of antifibrotic effects in KC treatment was confirmed in a curative approach using the daily administration of the antifibrotic agent NAC in the KC rat model (Figs 6 and 7). Fibrosis, defined by an abnormal accumulation of extracellular matrix components, is mediated by complex processes and is the endpoint of many chronic inflammatory disorders^23^,^34^,^43^. The initial stage of fibrosis is characterized by severe apoptosis due to tissue injury. This is followed by an inflammatory phase, wherein activated immune cells migrate into the tissue and release a variety of cytokines, including TNF-α^44^. Finally, abnormal accumulation of extracellular matrix components with pathological features leads to organ malfunction, and sustained activation of TGF-β signaling is involved in the unbalanced accumulation of the extracellular matrix^34^,^45^. The administration of MSCs to the KC bladder significantly blocked the apoptosis, mast-cell infiltration (Fig. 3a,b), upregulation of inflammatory cytokines such as TNF-α, and activation of TGF-β signaling (Fig. 5). These results suggest that MSCs may exert protective effects in each of these phases during the fibrotic changes of the KC bladder.

Several studies have shown that mast-cell infiltration is a critical determinant of the immune response implicated in KC^46^ and IC^47^,^48^. Because mast-cells, which are activated by allergic reactions^49^, release vasoactive intestinal peptide and TNF-α^48^,^49^, the infiltrated mast-cells induce the apoptosis and fibrosis of the KC bladder. As mentioned above, MSCs helped to relieve the mast-cell infiltration and consequent increase in pro-inflammatory cytokine transcripts in the KC bladder tissues. MSCs exert anti-inflammatory and immunomodulatory properties via cell contact and the secretion of soluble factors such as CD25 (IL2-R), CD38, CD69, IL-10, interferon-γ, indoleamine 2,3-dioxygenase, prostaglandin E2, and thrombospondin-2^12^,^19^,^24^,^25^,^50^. The value of these immunomodulatory effects has been shown in preclinical and clinical studies of several immunological disorders, including rheumatoid arthritis, multiple sclerosis, systemic lupus erythematosus, Crohn’s disease, and type 2 diabetes^51^,^52^,^53^,^54^,^55^. In the present KC animal model, most injected MSCs were distributed in the lamina propria near the basal layer of the urothelium (Fig. 4), suggesting that MSCs could protect against KC-induced injury by secreting certain paracrine factors that disrupt signaling pathways, leading to tissue inflammation and fibrosis. In addition, administration of MSCs may help to replace stromal and epithelial cells (Fig. 4b), which play an important role in maintaining urothelium integrity^56^ and are targets of the apoptotic signals induced in the bladder by KC (Fig. 3c). However, the spare distribution of injected GFP^+^ MSCs mainly in the lamina propria (Fig. 4a) is not sufficient to explain the anti-fibrotic effect throughout the whole bladder (Fig. 3c) by a single local administration of MSCs. Therefore, identification of the precise mechanism or key paracrine factor(s) of the immunomodulatory and anti-fibrotic effects of MSC therapy on KC could advance not only the efficacy of stem cell therapy, but also our understanding of the pathophysiology of KC.

The present study showed that an MSC-based therapy can exert pleiotropic effects in KC treatment and elucidated the mechanism underlying the MSC therapy. These promising results could overcome the skepticism surrounding MSC therapy regarding the uncertain explanation of the mechanisms involved and the poor engraftment and long-term *in vivo* survival of the administered MSCs^14^. Therefore, to successfully translate promising preclinical studies into clinical practice, several points should be assessed. Optimization of the MSC dose and timing of the administration and the identification of cost-effective and efficacious patient-specific stem cell sources should be considered to develop an efficacious “gold standard” stem cell approach that can be used to treat intractable urological disorders such as KC in the near future. Furthermore, the priority in the clinic will be critical assessment of the safety of stem cell therapy.

In conclusion, our findings provide the first evidence of the therapeutic effect of MSCs in KC and elucidation of the mechanism underlying the pleiotropic effects of MSCs that protect against fibrotic changes, as well as mast-cell infiltration and apoptosis, in the KC bladder. Furthermore, the significance of the antifibrotic effect was confirmed in a pharmacotherapy-based approach. Therefore, these promising preclinical results raise hopes that a therapeutic approach for KC patients could be found in upcoming clinical trials using MSCs.

## Methods

### KC rat model

All animal experiments were approved by and performed in accordance with the guidelines of the Institutional Animal Care and Use Committee of the University of Ulsan College of Medicine (IACUC-2013-12-134). To induce KC, 10-week-old female Sprague-Dawley rats (Orient Bio, Gapyong, Gyeonggi-do, Korea) were administered 25 mg/kg ketamine hydrochloride (EK1352-11; Huons, Seongnam-si, Gyeonggi-do, Korea) via intravenous injection daily for 5 days, followed by a 2-day rest period, in each of two cycles. A dosage of ketamine was selected on the basis of our preliminary experiments and the induction of KC was validated by conscious cystometry and histological analysis prior to injection of MSCs (Supplementary Fig. 5). For the sham group (*n* = 10), phosphate-buffered saline (PBS) was injected instead of ketamine. Two days after the last ketamine injection, an abdominal incision was made and UCB-derived MSCs at a density of 1 × 10^6^ cells per 200 μl PBS (KC + MSC group; *n* = 10) or PBS vehicle (KC group; *n* = 10 and sham group) were directly injected into the submucosal layer of either the anterior wall or dome of the bladder using a 500-μm syringe and 26-gauge needle. One week after MSC injection, the therapeutic outcomes of the MSCs were examined by conscious cystometry, histological analysis, and gene expression analysis (Fig. 1). In addition, to prevent tissue fibrosis, 200 mg/kg NAC (Sigma-Aldrich, St. Louis, MO) was administered into a group of ketamine-injected rats via daily intraperitoneal injection for 5 days, followed by a 2-day rest period (KC + NAC group, *n* = 5). For control, same volume (500 μl) of PBS without NAC was injected into sham and KC groups (*n* = 5).

### Cultivation of human UCB-derived MSCs

Human UCB was obtained from healthy, normal, full-term newborns after obtaining written informed parental consent in accordance with the guidelines approved by the Ethic Committee on the Use of Human Subjects at MEDIPOST Co., Ltd. (Seongnam-si, Gyeonggi-do, Korea). Informed consent was obtained from all pregnant mothers before UCB collection. Human UCB-derived MSCs used in this study were provided by MEDIPOST Co., Ltd. and grown in high-glucose Dulbecco’s modified Eagle’s medium (HyClone, Pittsburgh, PA) supplemented with 2 mM L-glutamine, 20 mM HEPES (pH 7.3), MEM nonessential amino acid solution, penicillin/streptomycin (Corning Cellgro, Pittsburgh, PA), 1 mg/mL ascorbic acid (Sigma-Aldrich), 10% heat-inactivated fetal bovine serum (HyClone), 5 ng/mL human epidermal growth factor (Sigma-Aldrich), 10 ng/mL basic fibroblast growth factor, and 50 mg/mL Long R3 insulin-like growth factor-1 (ProSpec, Rehovot, Israel) in a humidified atmosphere with 5% CO_2_ at 37 °C. MSCs expanded less than six passages were used for transplantation to ensure their multipotency. Basic characteristics of MSCs were examined by expression of surface antigen characterization (positive staining of CD29, CD73, and CD105, but lack of expression for CD34 and CD45 hematopoietic lineage markers) as well as multipotent differentiation capacity which are evidenced by *in vitro* differentiation into osteogenic, chondrogenic, or adipogenic lineages (Supplementary Fig. 6), as previously described^57^. To establish MSCs stably expressing GFP protein, cells were infected with lentivirus containing a *GFP*-expressing cassette in the pLenti6.2/V5-DEST Gateway lentiviral vector (Invitrogen), followed by the maintenance of cells in culture media supplemented with 6 μg/ml Blasticidin (Invitrogen), according to the manufacturer’s instructions.

### Cystometry

Cystometry was performed by first placing the conscious rats under restraints. They were then anesthetized and subjected to a midline suprapubic incision to expose the bladder. The bladder was accessed by using an inflatable polyethylene-50 tube (Clay Adams, Parsippany, NJ) connected to a pressure transducer. Sterile saline was infused at a rate of 40 μL/min via a micro-infusion pump (Harvard Apparatus, Holliston, MA). The urodynamics were measured by using the UDS-120XLT urodynamic measurement system (Laborie Medical Technologies, Toronto, Canada). Intravesical pressure was recorded by using a pressure analyzer and a computer-based data acquisition system. Voiding contraction was defined as an increase in bladder pressure that resulted in urine loss. The maximum contraction pressure (mmHg) was measured at peak pressure for contraction. Detrusor overactivity was diagnosed when an irregularly increasing frequency of contraction was observed. Bladder capacity was defined by the sum of the voiding volume and residual urine volume.

### Histo- and immunohistochemical analyses

After 24-hours fixation in 4% paraformaldehyde, each bladder was embedded in paraffin, cut on a microtome into 3-μm-thick slices, affixed to slides, and stained with hematoxylin and eosin. Mast-cell infiltration and fibrosis were assessed by Toluidine blue staining (Toluidine blue-O; Daejung Chemicals & Metals, Seoul, Korea) and Masson’s trichrome staining (Junsei Chemical, Tokyo, Japan), respectively. To measure apoptosis, the bladder was stained with antibodies specific for TUNEL (Roche, Mannheim, Germany), followed by visualization using Alexa 488-conjugated anti-mouse or -rabbit antibodies (Molecular Probes, Grand Island, NY). Tracking of injected MSCs was performed by detecting the fluorescent signal of GFP in the bladder. The epithelial and stromal characteristics of GFP^+^ cells were further examined by staining with antibodies against β-catenin (Cell Signaling Technology, Danvers, MA) and vimentin (Santa Cruz Biotechnology, Santa Cruz, CA), respectively. The status of transforming growth factor-beta (TGF-β) signaling was examined by immunostaining of phosphorylated Smad2 (Ser465/467; Cell Signaling Technology) or Smad3 (Ser423/425; Cell Signaling Technology). The immunostaining was visualized using Alexa 488- or 564-conjugated anti-mouse or -rabbit antibodies (Molecular Probes). The nuclei were counterstained with 4′,6-diamino-2-phenylindole (DAPI). Quantitative digital image analysis was performed in seven randomly chosen representative areas from each slide. To quantify the fibrosis and apoptosis, the area positively stained with Masson’s trichrome staining and TUNEL assay were calculated using Image-Pro 5.0 software (Media Cybernetics, Rockville, MD). The mast-cell infiltration and activation of TGF-β signaling were quantified by counting the cells stained with Toluidine blue and immunostaining of phosphorylated Smad2 or Smad3, respectively.

### Reverse transcriptase and real-time quantitative PCR

The total RNA from the bladder tissues was isolated by using the RNeasy Mini Kit (QIAGEN, Valencia, CA). Genomic DNA was extracted by using the DNA-free Kit (Applied Biosystems, Foster City, CA). mRNA (400 ng) was reverse-transcribed by using TaqMan Reverse-Transcription Reagents (Applied Biosystems) according to the manufacturer’s instructions. Target gene expression was quantified by RQ-PCR by using the iQ5 Optical System (Bio-Rad, Hercules, CA) with iQ SYBR Green PCR Master Mix (Bio-Rad), as previously described^57^. All primers used in the RQ-PCR assay are available on request.

### Statistical analysis

Data were reported as the mean ± standard error of the mean (SEM) and were analyzed by GraphPad Prism 6.0 software (GraphPad Software, La Jolla, CA). Differences and significance were verified by one-way ANOVA followed by Bonferroni post hoc tests. A p-value less than 0.05 was considered statistically significant.

## Additional Information

**How to cite this article**: Kim, A. *et al.* Mesenchymal stem cells protect against the tissue fibrosis of ketamine-induced cystitis in rat bladder. *Sci. Rep.* **6**, 30881; doi: 10.1038/srep30881 (2016).

## Supplementary Material

Supplementary Information
