# Supplementary material for: Mesenchymal stem cells protect against the tissue fibrosis of ketamine-induced cystitis in rat bladder
Source: Sci Rep. 2016 Aug 2;6:30881. doi: 10.1038/srep30881 (PMC4969614; doi:10.1038/srep30881)
Supplement: Supplementary Information [file srep30881-s1.pdf]

Supplementary Information

Mesenchymal stem cells protect against the tissue fibrosis of ketamine-induced cystitis in rat bladder

Aram Kim[1,†], Hwan Yeul Yu[1,2,†], Jinbeom Heo[2,3,†], Miho Song[1], Jung-Hyun Shin[1], Jisun Lim[2,3], Soo-Jung Yoon[1,2], Yong Hwan Kim[2,3], Seungun Lee[2,3], Seong Who Kim[4], Wonil Oh[5], Soo Jin Choi[5], Dong-Myung Shin[2,3,*], Myung-Soo Choo[1,*]

[1]Department of Urology, [2]Department of Biomedical Sciences, [3]Department of Physiology, [4]Department of Biochemistry and Molecular Biology, Asan Medical Center, University of Ulsan College of Medicine, Seoul, Korea; [5]Biomedical Research Institute, MEDIPOST Co., Ltd., Seoul, Korea

[†]These authors contributed equally to this work.

Running title: MSC therapy for ketamine-induced cystitis

[*]**Correspondence:**

Myung-Soo Choo, MD, PhD, Department of Urology, Asan Medical Center, University of Ulsan College of Medicine, 88 Olympic-ro 43-gil, Songpa-gu, Seoul 05505, Korea

Tel: 82-2-3010-3735; Fax: 82-2-477-8928; Email: mschoo@amc.seoul.kr

Dong-Myung Shin, Ph.D., Department of Biomedical Sciences, Asan Medical Center, University of Ulsan College of Medicine, 88 Olympic-ro 43-gil, Songpa-gu, Seoul 05505, Korea

Email: d0shin03@amc.seoul.kr

Supplementary Figures and Figure Legends

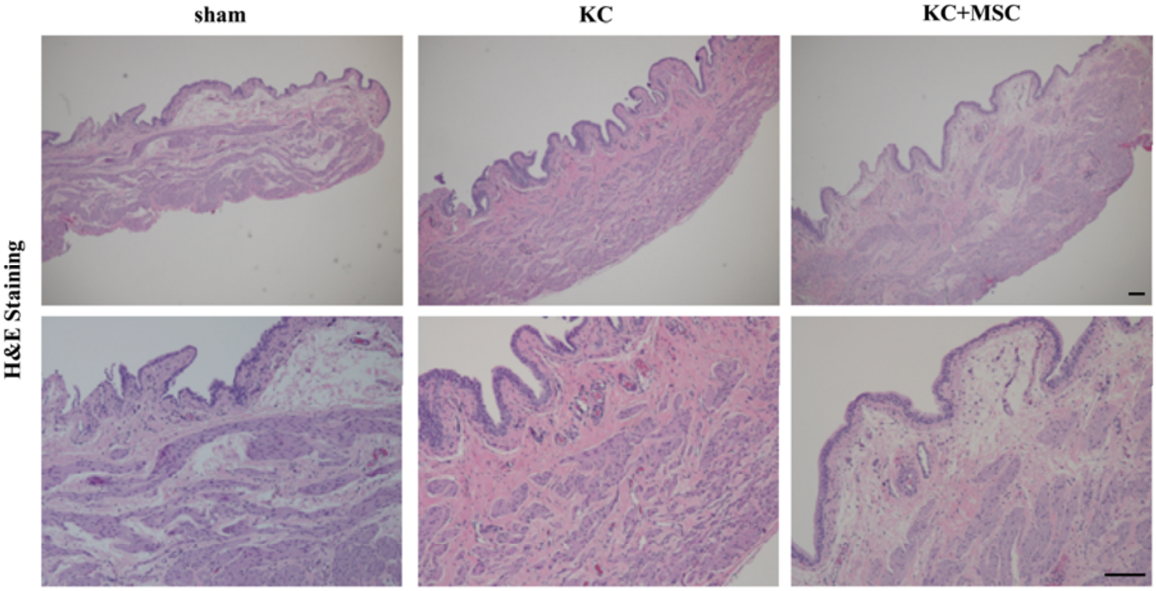

Supplementary Figure S1. Histological analysis of the KC bladder. Hematoxylin and eosin staining in the indicated bladder tissues. Nuclei were stained with Mayer's hematoxylin. The upper and lower images were at ×40 and ×100 magnification (scale bar = 100 μm), respectively. KC = ketamine-induced cystitis; MSC = mesenchymal stem cell.

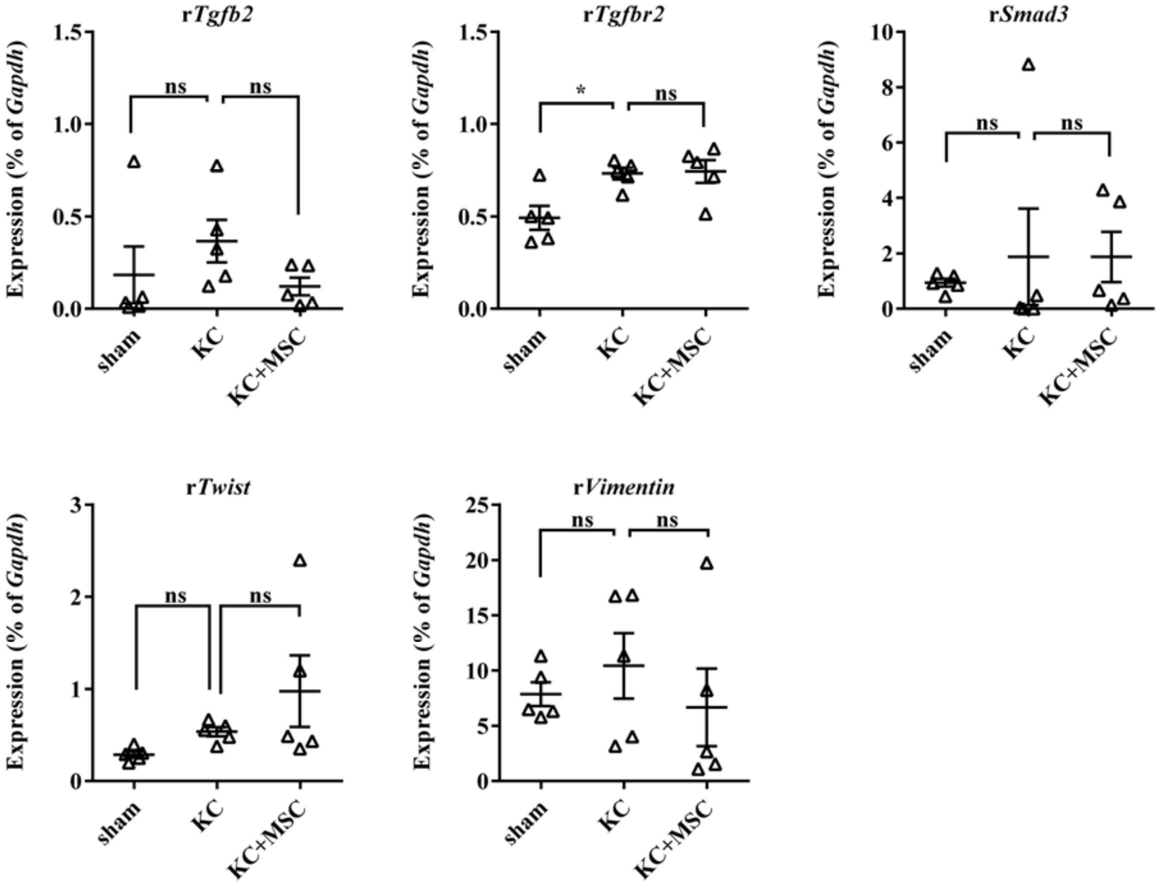

Supplementary Figure S2. RQ-PCR analysis of tissue fibrosis-related genes in the indicated bladder tissues. The expression level is presented as % *Gapdh* and shown as dot plot with the mean ± SEM, $n = 5$, *p<0.05 compared with the KC group with Bonferroni post-test. ns = non-significant.

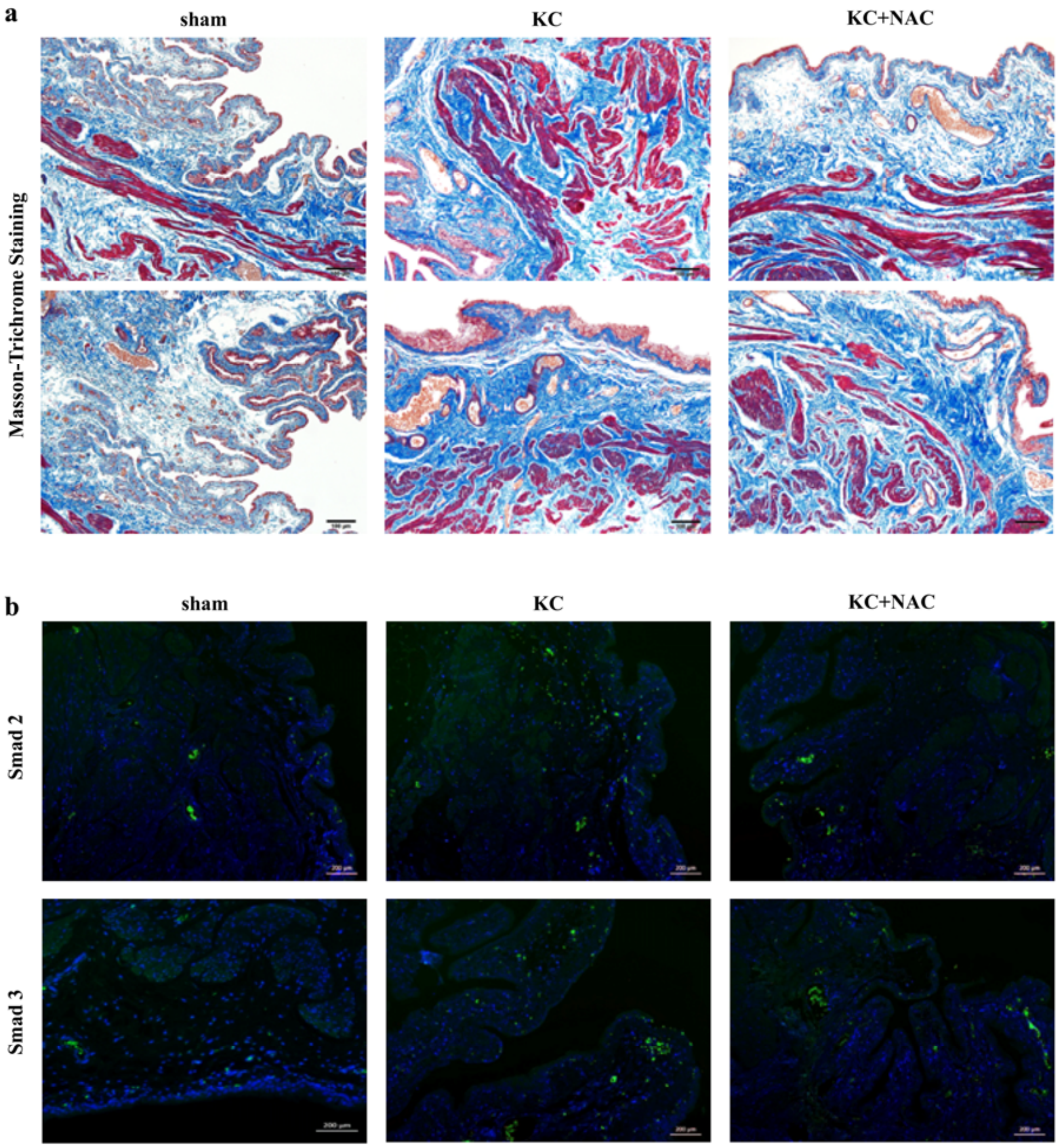

Supplementary Figure S3. The beneficial effects of NAC on fibrotic changes in the KC bladder. (a) Masson's trichrome staining in the indicated bladder tissues (magnification ×200). Scale bar = 200 μm. (b) Fluorescent immunostaining of phosphorylated Smad2 or Smad3 protein (green) at 2 d after the last injection of NAC (magnification ×100). Nuclei were stained with DAPI (blue). Scale bar = 200 μm. KC = ketamine-induced cystitis; NAC = N-acetylcysteine.

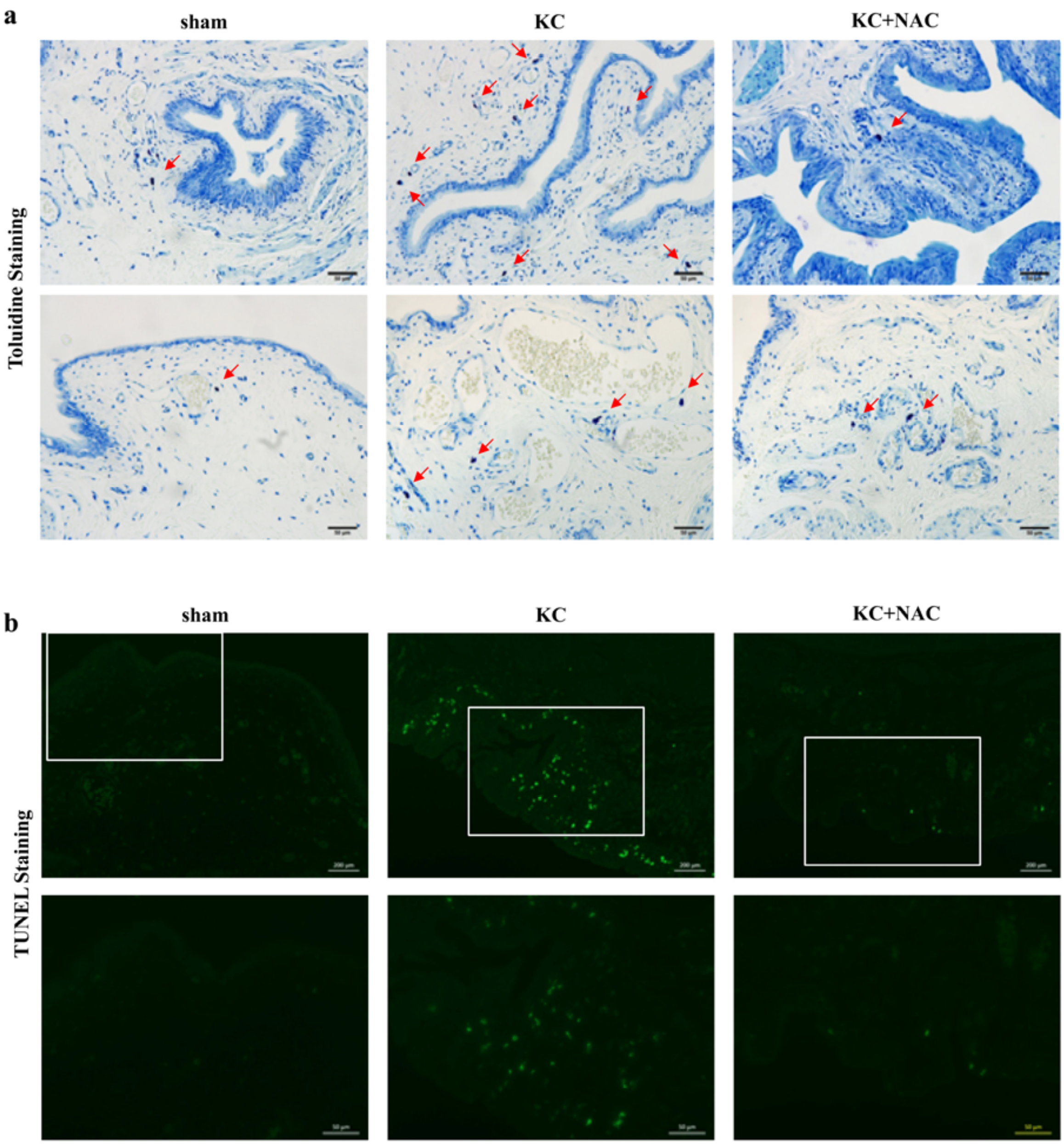

Supplementary Figure S4. NAC therapy ameliorated the histological abnormalities of the KC bladder. (a and b) Toluidine (a; magnification ×200, scale bar = 50 μm) and TUNEL (b) staining in the indicated bladder tissues. The region characterized by apoptotic cells (box in upper image; magnification ×100, scale bar = 200 μm) is shown in the lower panel at higher magnification (×200, scale bar = 50 μm).

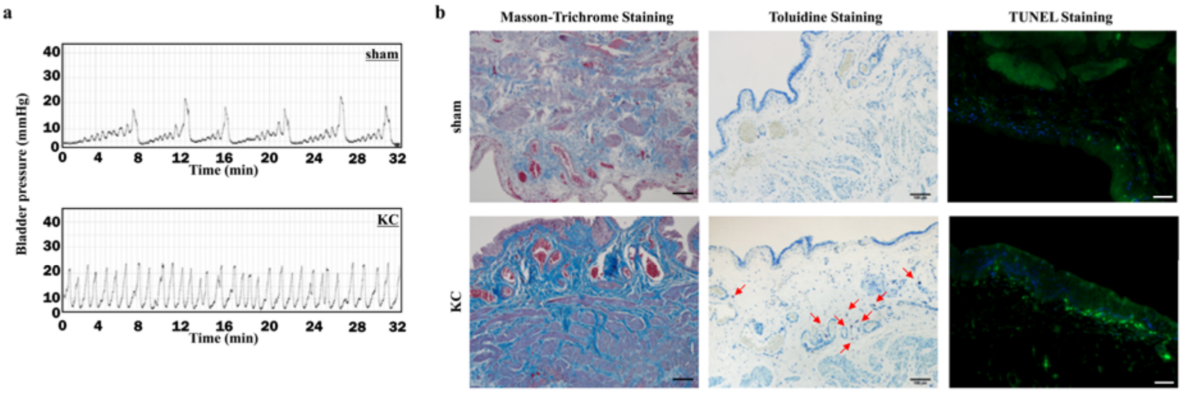

Supplementary Figure S5. Validation of KC animal model. (a) Representative conscious cystometry of the bladders 2 week after the initial the injection of 25 mg/kg ketamine prior to administration of human UCB-derived MSCs. (b) Histological analysis of ketamine-induced bladder injuries. (Left panel) Masson's trichrome staining (magnification ×100, scale bar = 100 μm). (Middle panel). Toluidine blue staining (magnification ×100, scale bar = 100 μm). (Right panel) a TUNEL assay to detect the apoptotic cells (green) in the bladder sections (magnification ×200, scale bar = 50 μm). Nuclei were stained with DAPI (blue). KC = ketamine-induced cystitis.

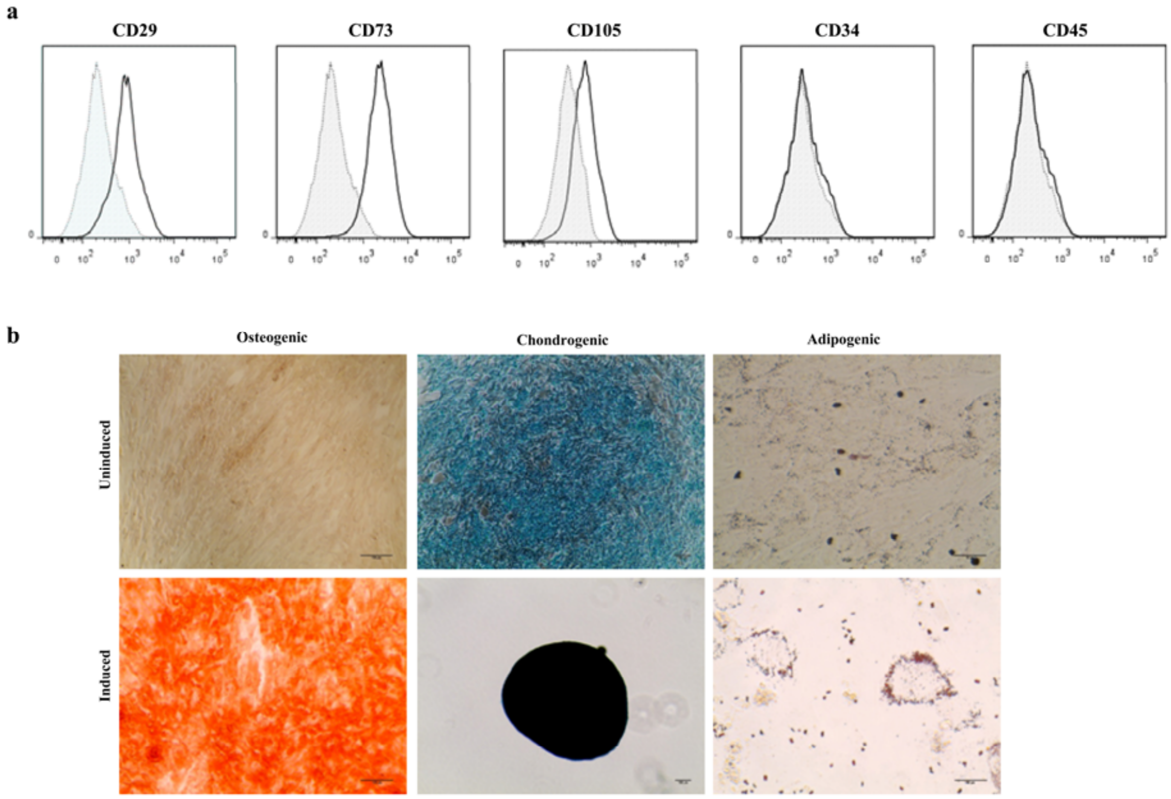

Supplementary Figure S6. Characterization of MSCs. (a) Flow cytometry analysis of the expression of the indicated MSC surface (CD29, CD73, and CD105) and hematopoietic (CD34, and CD45) lineage proteins in human UCB-derived MSCs used in this study. (b) Representative images of the osteogenic, chondrogenic, and adipogenic differentiation assays in which each lineage differentiation was determined using Alizarin Red S (magnification ×200, scale bar = 100 μm), Alcian Blue (magnification ×100, scale bar = 100 μm), and Oil Red O staining (magnification ×200, scale bar = 50 μm), respectively.
